# Mendelian randomization study updates the effect of 25-hydroxyvitamin D levels on the risk of multiple sclerosis

**DOI:** 10.1186/s12967-021-03205-6

**Published:** 2022-01-03

**Authors:** Renxi Wang

**Affiliations:** Beijing Institute of Brain Disorders, Laboratory of Brain Disorders, Ministry of Science and Technology, Collaborative Innovation Center for Brain Disorders, Capital Medical University, No.10 Xitoutiao, You An Men, Beijing, 100069 China

**Keywords:** Multiple sclerosis, 25OHD, Genome-wide association study, Mendelian randomization, Single nucleotide polymorphism

## Abstract

**Background:**

Observational studies and previous Mendelian randomization (MR) studies have shown that genetically low 25-hydroxyvitamin D (25OHD) levels are associated with a high susceptibility to multiple sclerosis (MS). The present MR study aims to update the causal estimates for the effects of 25OHD levels on MS risk.

**Methods:**

To date, the largest genome-wide association study (GWAS) for serum 25OHD (n = 401,460) and MS (14,498 MS cases and 24,091 controls) was used to assess the effect of serum 25OHD levels on MS. All participants were of European ancestry. The MR-egger_intercept test and Cochran’s Q statistic were used to determine the pleiotropy and the heterogeneity, respectively. MR-egger, weighted median, inverse variance weighted (multiplicative random effects), simple mode, and weighted mode methods were used to evaluate the causal association of serum 25OHD levels with MS. Finally, the effect of a single 25OHD SNP (single nucleotide polymorphism) on MS was used to test the SNP bias.

**Results:**

One hundred and fifteen newly identified serum 25OHD genetic variants were extracted from a large-scale serum 25OHD GWAS dataset. The 20 most effective and independent 25OHD genetic instrumental variables were extracted from the MS GWAS summary statistics. Pleiotropy analysis suggested no significant pleiotropic variant among the 20 selected 25OHD genetic instrument variants in MS GWAS datasets. As serum levels of 25OHD based on genetic changes increased, the risk of MS decreased using MR-egger (Beta = − 0.940, *p* = 0.001; OR = 0.391), weighted median (Beta = − 0.835, *p* = 0.000; OR = 0.434), IVW (Beta = − 0.781, *p* = 0.000; OR = 0.458), simple mode (Beta = − 1.484, *p* = 0.016; OR = 0.227), and weighted mode (Beta = − 0.913, *p* = 0.000; OR = 0.401). Our results were robust, with no obvious bias based on investigating the single 25OHD SNP on MS.

**Conclusions:**

Our analysis suggested a causal association between genetically increased serum 25OHD levels and reduced MS in the European population.

**Supplementary Information:**

The online version contains supplementary material available at 10.1186/s12967-021-03205-6.

## Background

Multiple sclerosis (MS) is an inflammatory autoimmune disease in the central nervous system [1–4]. The prevalence of MS is rising globally [1]. In the past decades, better understanding of the disease mechanisms of MS has led to several disease-modifying therapy development [5]. However, current therapeutic treatments for MS still remain disappointing [5]. It is necessary to explore the pathogenic mechanisms driving MS [5]. It is known that MS is mediated by the interaction between genetic and environmental factors and well-powered genome-wide association studies (GWAS) have been explored to investigate the genetic risk factors in MS [6].

The active form of vitamin D, 1,25-Dihydroxyvitamin D3 (1,25(OH)2D3), suppresses autoimmune diseases such as MS by reducing the production of proinflammatory cytokines such as IFN (interferon)-γ, IL (interleukin)-2, and IL-17, as well as enhancing the secretion of anti-inflammatory cytokines such as IL-4 and IL-10 [7–11]. A recent retrospective case–control study showed that the effect of vitamin D3 on MS susceptibility was not mediated by regulatory T cells (Tregs) [12]. Increased evidences suggest that decreased vitamin D levels is associated with an abnormal immune response in MS [13, 14]. Low vitamin D levels have been shown to correlate with disease activity in various autoimmune disorders and neurological diseases including MS [13–19]. Numerous epidemiological studies have strongly suggested that vitamin D insufficiency contributes to MS risk [20]. One SNP, rs10766197 CYP2R1 which regulates serum 25-OH-vitamin D3 (25OHD) levels, is reported to relate with MS risk [21, 22]. Thus, hypovitaminosis D was considered as a possible risk factor for MS, with multiple decades of research on the association of vitamin D status and MS [23].

Mendelian randomization (MR) studies have been widely used to assess the causal link between an exposure and an outcome by an analytical method using genetic variants as instrumental variables [13, 24–26]. Four previous MR studies used three or four serum 25OHD genetic instrumental variables (IVs) and suggested a link between predicted serum 25OHD and the risk of MS [27–30]. MR studies have many limitations, including low statistical power. Thus, to improve statistical power, 20 serum 25OHD genetic IVs from the largest GWAS for serum 25OHD (n = 401,460) were used to update the effect of serum 25OHD levels on MS risk.

## Methods

### Ethics approval and consent to participate

Our study was approved by the Ethics Committee of Beijing Institute of Brain Disorders in Capital Medical University. This article contains human participants collected by several studies to report the large-scale GWAS for the serum 25OHD [31] and MS [32]. All participants gave informed consent in all the corresponding original studies, as described in the Methods.

### Study design

**Fig. 1:**
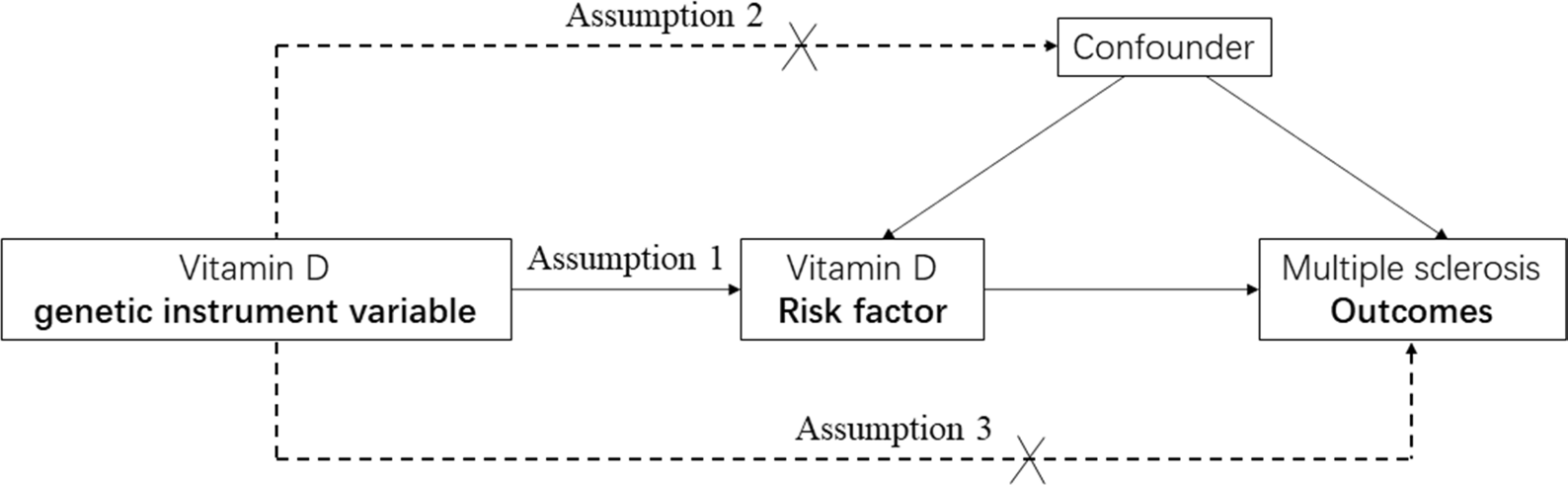
Design flow chart for the MR study. MR assumptions: assumption 1, 2 and 3. Solid line represents direct causal effects that 25OHD genetic instrument variants are reliably associated with 25OHD levels and influence the risk of MS through the 25OHD in assumption 1. The dotted line represents that 25OHD genetic instrument variants are not associated with any measured or unmeasured confounders and do not influence the risk of MS through other pathways in assumption 2 and 3, respectively

Three principal assumptions were prerequisites in the MR study [24, 25] and are described in Fig. 1. Assumption 1 is that genetic instrument variants are reliably associated with serum 25OHD levels. Assumption 2 is that genetic instrument variants should undoubtedly not be associated with any confounders. Assumption 3 is that genetic instrument variants firmly influence the risk of MS through the 25OHD and not through other pathways.

### Genetic instrument variants for serum 25OHD

Sixty-nine independent 25OHD genetic variant loci were identified using imputed genotypes from 401,460 white British UK Biobank participants with available serum 25OHD levels [31]. The TwoSampleMR R package was used to perform a two-sample MR study. One hundred and fifteen independent 25OHD genetic variants with each trait at *p* < 5 × 10^−8^ are demonstrated in Additional file 1: Table S1 and were selected as “suggestive” instruments in our MR studies, as described in recent studies [24, 25]. To obtain a more accurate effect size estimate with these variants, the following rules was used to remove some variants. (1) Variants with a linkage disequilibrium (LD) (r^2^ > 0.05) were removed by using LDlink (https://ldlink.nci.nih.gov/?tab=ldmatrix, CEU). (2) Palindromic variants resulting in potential strand ambiguity were removed. (3) To ensure unconfounded instruments only affected MS via the relevant exposure, variants associated with possible exposure-outcome confounders (e.g. age, smoking, and socioeconomic position) were also removed. Finally, 20 effective and independent 25OHD-associated single nucleotide polymorphisms (SNPs) were selected as the potential IVs and are shown in Table 2.

### MS GWAS dataset

The MS GWAS dataset is from the International Multiple Sclerosis Genetics Consortium (IMSGC) and consists of 14,498 MS subject and 24,091 healthy control individuals of European ancestry [32]. The TwoSampleMR R package was used to perform a two-sample MR study. Twenty effective and independent 25OHD genetic variants were extracted from MS GWAS dataset (https://gwas.mrcieu.ac.uk/datasets/ieu-a-1025/) by using functions “extract_outcome_data” and “harmonise_data”. Table 1 provides the sample profiles for the MS GWAS datasets.

**Table 1:** The sample information in the MS GWAS datasets

Population	Sex	MS cases	Control	Number of SNPs
European	Males and females	14,498	24,091	156,632

### Pleiotropy test

The MR-egger_intercept test was used to determine the pleiotropy test [33]. The TwoSampleMR R package was used to perform the MR-egger_intercept test. The MR-egger_intercept test was performed using function “mr_pleiotropy_test”. *P* < 0.05 is the level of statistical significance.

### Heterogeneity test

Cochran’s Q statistic was used to determine the heterogeneity test [34, 35]. The TwoSampleMR R package was used to perform Cochran’s Q statistic. The heterogeneity test was performed by using function “mr_heterogeneity”. *P* < 0.05 is the level of statistical significance.

### MR analysis

The TwoSampleMR R package was used to perform the MR analysis. The MR analysis was performed by using function “mr”. We selected five MR analysis methods including MR-egger, weighted median, inverse variance weighted (multiplicative random effects), simple mode, weighted mode [33, 36, 37]. The effect size (beta) and 95% confidence interval (CI) correspond to 1 standard deviation (SD) in 25OHD levels. *P* < 0.05 is the level of statistical significance.

### Single SNP effect analysis

The TwoSampleMR R package was used to analyze the effect of serum single 25OHD SNP on MS. This analysis was performed by using functions “mr_singlesnp” and “mr_leaveoneout”. Forest plot were prepared by using functions “mr_forest_plot” and “mr_leaveoneout_plot”.

## Results

### Serum 25OHD genetic instrument variants in MG GWAS dataset

To explore the effect of serum 25OHD genetic instrument variants on MS, we used the MS GWAS dataset (https://gwas.mrcieu.ac.uk/datasets/ieu-a-1025/), described in Table 1, to extract 20 effective and independent 25OHD genetic variants from the MS GWAS dataset by using functions “extract_outcome_data” and “harmonise_data”. Table 2 showed the detailed information about 20 25OHD genetic instrument variants in the MS GWAS dataset.

**Table 2:** Association of 20 vitamin D genetic variants in MS GWAS dataset

SNP	Serum 25OHD GWAS			MS GWAS		
	EA	NEA	EAF	Beta	SE	*p* val
rs8063565	C	G	0.733	− 0.002	0.019	0.928
rs11542462	A	G	0.130	0.026	0.025	0.292
rs964184	C	G	0.134	− 0.014	0.024	0.570
rs1260326	C	T	0.601	0.005	0.017	0.749
rs1800588	T	C	0.219	− 0.005	0.020	0.786
rs10908469	C	A	0.278	− 0.021	0.018	0.262
rs1532085	G	A	0.607	− 0.006	0.017	0.717
rs2131925	G	T	0.318	0.005	0.020	0.805
rs7528419	A	G	0.784	0.025	0.020	0.225
rs804281	G	A	0.576	0.028	0.017	0.098
rs532436	A	G	0.210	0.042	0.020	0.034
rs10822145	T	C	0.472	− 0.014	0.016	0.398
rs1660818	A	G	0.317	0.000	0.018	0.999
rs2276360	C	G	0.785	− 0.105	0.018	0.000
rs12317268	G	A	0.162	0.037	0.022	0.084
rs11182428	C	T	0.514	0.017	0.017	0.302
rs61003750	C	G	0.300	0.029	0.018	0.122
rs142158911	A	G	0.120	− 0.071	0.026	0.006
rs62115743	T	C	0.077	− 0.056	0.031	0.071
rs1841850	C	A	0.114	− 0.051	0.025	0.040

*SNP* single-nucleotide polymorphism, *EA* effect allele, *NEA* non-effect allele, *EAF* effect allele frequency; Beta is the regression coefficient based on the vitamin D raising effect allele, *SE* standard error

### Pleiotropy analysis

MR-egger_intercept test was performed to determine the pleiotropy test by using function “mr_pleiotropy_test”. Table 3 shows detailed information about the pleiotropy analysis of 20 25OHD genetic instrument variants in MG GWAS dataset. The statistical *P* = 0.409 suggests that there is no significant pleiotropic variant among the selected 20 25OHD genetic instrument variants in the MS GWAS datasets. Thus, we established the assumption that genetic instrument variants should not be associated with any confounders. Therefore, all these 20 25OHD genetic variants could be used as the effective instrumental variables in this MR analysis.

**Table 3:** Pleiotropy test of 20 serum 25OHD genetic variants in MS GWAS dataset

GWAS dataset	MR-Egger intercept		
	Egger_intercept	SE	p-val
MS	0.007	0.008	0.409

*SE* standard error. p < 0.05 is set as the significant threshold. p-val = 0.409 represents no significant pleiotropy

### Heterogeneity test

Cochran’s Q statistic was performed to test the heterogeneity by using function “mr_heterogeneity”. Table 4 shows detailed information about the heterogeneity analysis of 20 25OHD genetic instrument variants in the MS GWAS dataset. Both MR-egger and inverse variance weighted methods demonstrated the statistical *P* = 0.04. *P* < 0.05 suggests significant heterogeneity among the 20 25OHD genetic instrument variants in MG GWAS datasets. Therefore, we mainly use the random effect model to estimate the MR effect size.

**Table 4:** Heterogeneity test of 20 serum 25OHD genetic variants in MS GWAS dataset

Method	MR-Egger intercept		
	Q	Q_df	Q_pval
MR Egger	29.446	18	0.043
Inverse variance weighted	30.614	19	0.044

p < 0.05 is set as the significant threshold. Q_pval = 0.04 represents significant heterogeneity

### MR analysis

**Fig. 2:**
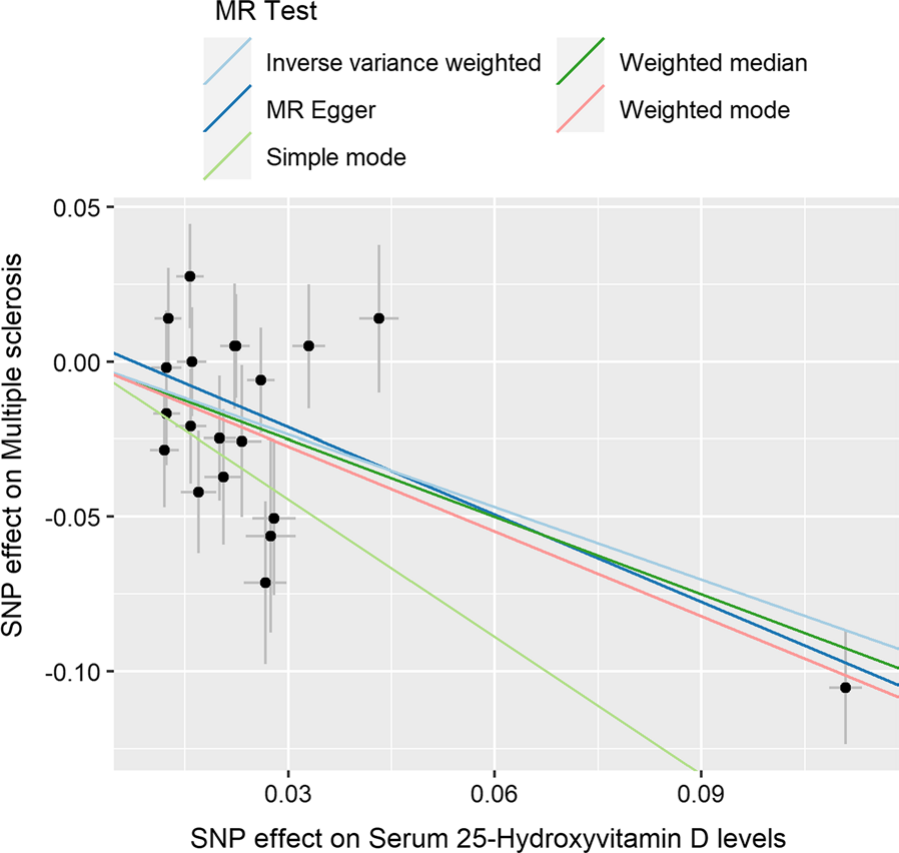
Individual estimates about the causal effect of serum 25OHD levels on MS. The x-axis shows the SNP (single nucleotide polymorphism) effect and SE (standard error) on each of the 20 serum 25OHD SNPs from 25OHD GWAS dataset (https://gwas.mrcieu.ac.uk/datasets/ebi-a-GCST90000615/). The y-axis shows the SNP effect and SE on multiple sclerosis (MS) from MS GWAS dataset (https://gwas.mrcieu.ac.uk/datasets/ieu-a-1025/). The regression lines for the MR-egger, weighted median, inverse variance weighted (multiplicative random effects), simple mode, weighted mode method are shown

We found that as 25OHD levels increased due to genetic differences, the risk of MS decreased. We used MR-egger (Beta = − 0.940, *p* = 0.001; OR = 0.391), weighted median (Beta = − 0.835, *p* = 0.000; OR = 0.434), IVW (Beta = − 0.781, *p* = 0.000; OR = 0.458), simple mode (Beta = − 1.484, *p* = 0.016; OR = 0.227), and weighted mode (Beta = − 0.913, *p* = 0.000; OR = 0.401) (Table 5). The individual MR estimates demonstrate that as the effect of a single SNP on 25OHD increased, the suppressive effect of a single SNP on MS increased, as determined using five MR analysis methods including MR-egger, weighted median, inverse variance weighted (multiplicative random effects), simple mode, and weighted mode (Fig. 2). Collectively, our data suggest causal association of increased 25OHD levels from greater genetic variation with decreased risk of MS.

**Table 5:** The causal association of serum 25OHD levels with MS

Method	nsnp	Beta	SE	*p* val	OR	OR_lci95	OR_uci95
MR Egger	20	− 0.940	0.251	0.001	0.391	0.239	0.639
Weighted median	20	− 0.835	0.162	0.000	0.434	0.316	0.596
IVW	20	− 0.781	0.165	0.000	0.458	0.331	0.633
Simple mode	20	− 1.484	0.560	0.016	0.227	0.076	0.679
Weighted mode	20	− 0.913	0.165	0.000	0.401	0.290	0.554

*IVW* Inverse variance weighted. IVW was based on multiplicative random effects. *nsnp* the number of single-nucleotide polymorphism. Beta: the regression coefficient based on 25OHD raising effect allele. SE: standard error. p < 0.05 represents the causal association of serum 25OHD levels with MS. *OR* Odds ratio. *OR_lci95* Lower limit of 95% confidence interval for OR. *OR_uci95* Upper limit of 95% confidence interval for OR

### Single SNP effect analysis

**Fig. 3:**
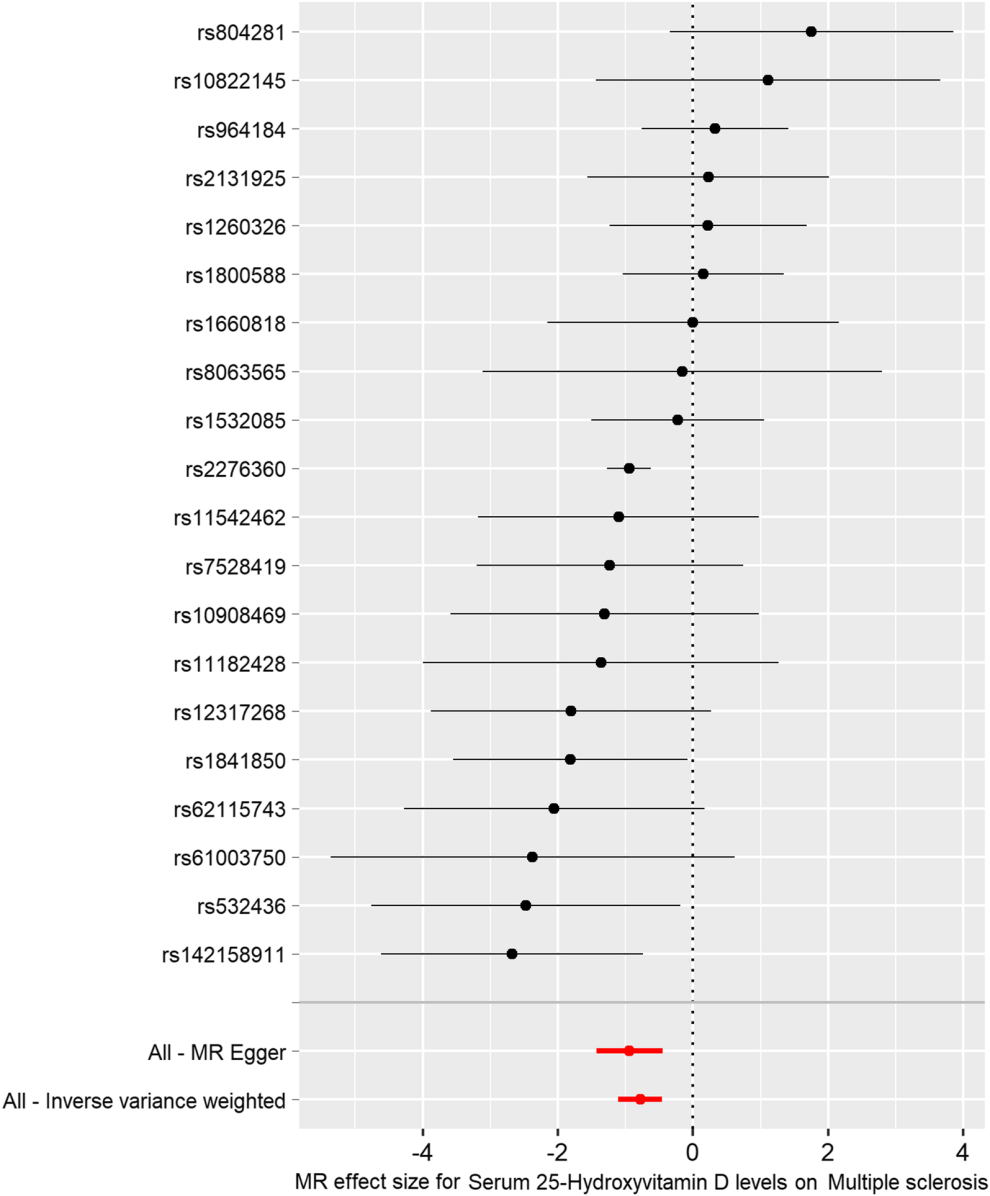
Forest plot of the 20 serum 25OHD SNPs associated with risk of MS. The x-axis shows the MR effect size for the 25OHD GWAS dataset (https://gwas.mrcieu.ac.uk/datasets/ebi-a-GCST90000615/) on multiple sclerosis (MS) from the MS GWAS dataset (https://gwas.mrcieu.ac.uk/datasets/ieu-a-1025/). The y-axis shows the analysis for each of the SNPs and for the SNPs in total using the MR-egger and inverse variance weighted (multiplicative random effects) methods

**Fig. 4:**
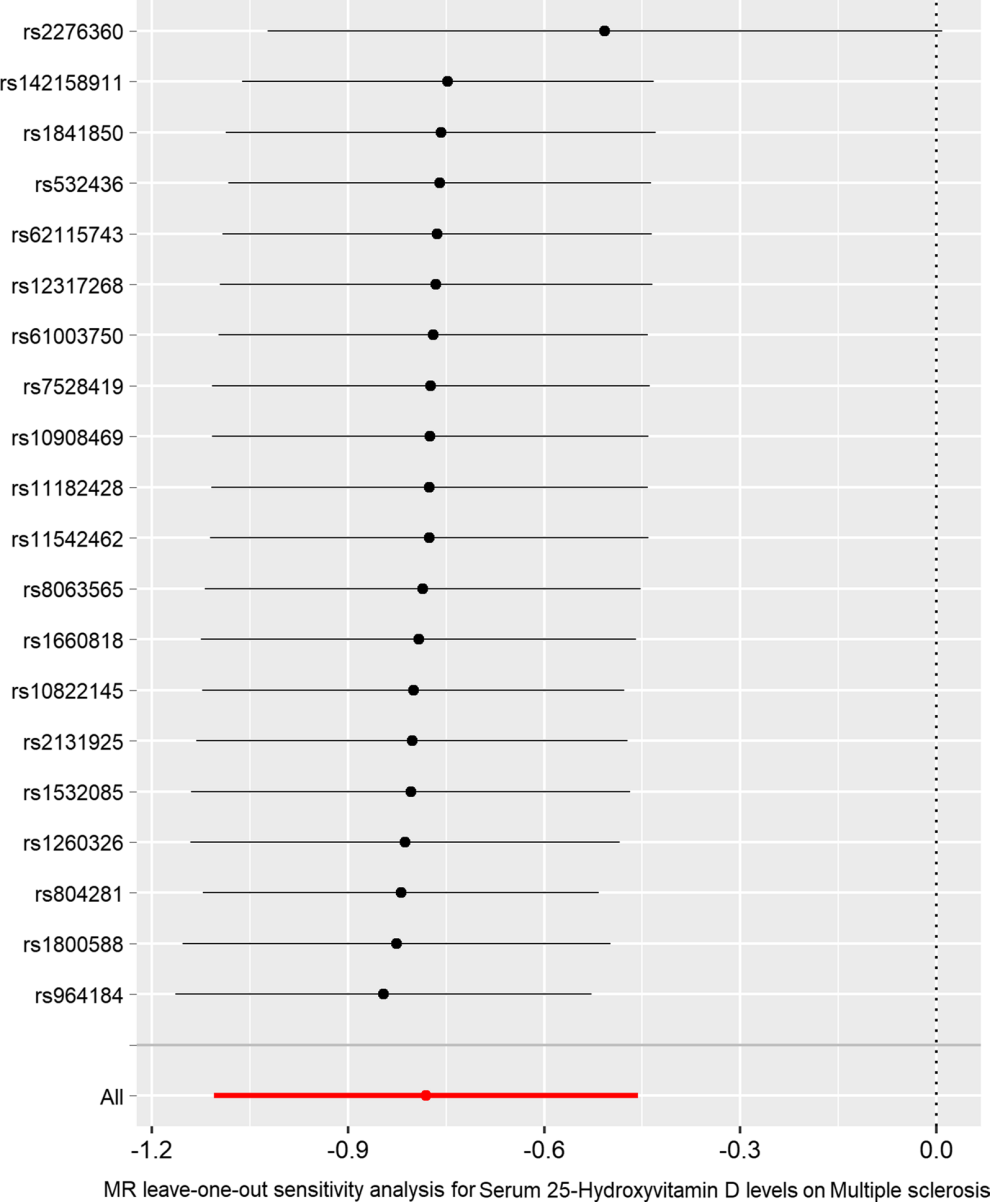
MR leave-one-out sensitivity analysis for the effect of the 20 serum 25OHD SNPs on MS. The x-axis shows the MR leave-one-out sensitivity analysis for serum 25OHD GWAS dataset (https://gwas.mrcieu.ac.uk/datasets/ebi-a-GCST90000615/) on multiple sclerosis (MS) from the MS GWAS dataset (https://gwas.mrcieu.ac.uk/datasets/ieu-a-1025/). The y-axis shows the analysis for leave-one-out of SNPs and the effect of the total SNPs on MS

A single effect of serum 25OHD SNP was performed by using functions “mr_singlesnp” and “mr_leaveoneout”. Forest plots were prepared by using functions “mr_forest_plot” and “mr_leaveoneout_plot”. A forest plot of the 20 serum 25OHD SNPs associated with risk of MS is demonstrated in Fig. 3. MR leave-one-out sensitivity analysis for the effect of the 20 serum 25OHD SNPs on MS is shown in Fig. 4. These findings indicate that our results are robust and have no obvious bias based on the effect of the single 25OHD SNP on MS.

## Discussion

Epidemiological evidence suggests an association between 25OHD deficiency and risk and disease progression of MS [13, 20, 38–44]. This relationship is further proven by four previous MR studies using three or four serum 25OHD genetic IVs [27–30]. In the first MR study in 2015, Mokry et al. [28] identified an odds ratio [OR] of 2 by using four serum 25OHD genetic IVs obtained from a serum 25OHD GWAS (N = 33,996) [45], as well as a MS GWAS involving 14,498 MS cases and 24,091 healthy controls from International Multiple Sclerosis Genetic Consortium (IMSGC) [32]. In the second MR study in 2016, Rhead et al. [29] identified an OR = 0.79 using three serum 25OHD genetic IVs. These samples were obtained from a serum 25OHD GWAS (N = 4501) [46] and MS GWAS from members of Kaiser Permanente Medical Care Plan, Northern California Region (KPNC) (1,056 MS cases and 9,015 controls) and the Epidemiological Investigation of Multiple Sclerosis and Genes and Environment in Multiple Sclerosis MS case–control studies (6335 cases and 5762 controls). In the third MR study in 2020, Jacobs et al. [27] identified an OR = 0.57 using six serum 25OHD genetic IVs obtained from a serum 25OHD GWAS (N = 79,366) [47] and IMSGC discovery phase GWAS (14,802 MS, 26,703 controls). In the fourth MR study in 2021, Harroud et al. [30] identified an OR = 0.72 using four serum 25OHD genetic IVs obtained from a serum 25OHD GWAS (N = 33,996) and IMSGC discovery phase GWAS (14,802 MS, 26,703 controls). In the present study, we identified an OR = 0.22 ~ 0.45 using 20 serum 25OHD genetic IVs from to date, the largest serum 25OHD GWAS (n = 401,460) [31] and MS GWAS (14,498 MS cases and 24,091 controls) [32]. Thus, our MR analysis further strengthens evidence for a causal link between 25OHD levels and MS risk.

Among 115 newly identified serum 25OHD genetic variants, 20 effectively independent 25OHD genetic instrumental variables were extracted from MS GWAS summary statistics. We did not identify any significant pleiotropic variant among the selected 20 25OHD genetic instrument variants in the MS GWAS datasets by pleiotropy analysis. Of note, five MR analysis methods demonstrated that 25OHD genetic instrument variants firmly influence the risk of MS through 25OHD but not through other pathways. We found that increased 25OHD levels due to genetic changes were significantly associated with reduced MS risk. For every 1 SD, genetically increased 25OHD levels could reduce MS risk 0.940, 0.835, 0.781, 1.484, and 0.913 using MR-egger, weighted median, inverse variance weighted (multiplicative random effects), simple mode and weighted mode, respectively. These results suggest a causal association between genetically increased 25OHD levels and MS risk.

Both MR-egger and inverse variance weighted methods demonstrated a significant heterogeneity among the 20 25OHD genetic instrument variants in the MS GWAS datasets. Thus, we mainly used the random effect model to estimate the MR effect size. Inverse variance weighted (multiplicative random effects) showed a reduced trend of MS with 1 SD genetically increased 25OHD levels (beta = − 0.781, 95% CI: [− 1.105, − 0.457], *p* = 2.34E−06). MR-egger, weighted median, simple mode and weighted mode also proved a causal link between 25OHD levels and MS risk. In addition, a single SNP effect analysis and leave-one-out sensitivity analysis for the effect of the 20 serum 25OHD SNPs on MS suggests that our results are robust with no obvious bias based on the effect of the single 25OHD SNP on MS.

25OHD deficiency is becoming an increasing problem worldwide. A recent finding led to a proposed link between 25OHD deficiency and autoimmune diseases [48]. It is reported that 25OHD deficiency is associated with MS risk [39]. Apart from the maintenance of healthy calcium metabolism, Vitamin D and its active product (1,25-dihydroxyvitamin D) have additional roles in immune and central nervous system cell homeostasis [38].

To date, there is evidence to suggest there are some benefits of vitamin D for patient-important outcomes among people with MS [40]. However, vitamin D did not affect recurrence of relapse and worsening of disability in patients with MS [40]. However, in clinical trials, while doses of up to 40,000 IU/day were tested and appeared safe as add-on therapy for short-term periods, chronic and high-dose therapy can lead to life-threatening complications related to vitamin D toxicity, including renal failure, cardiac arrythmia and status epilepticus [43].

There were several strengths to this MR study. First, this MR design was based on three principal assumptions and we used different methods to prove three principal assumptions. Second, we used the large-scale serum 25OHD GWAS dataset (n = 401,460) and MS GWAS summary statistics (14,498 MS cases and 24,091 controls) [31, 32]. With a large-scale GWAS, it is easy to find a causal association between 25OHD levels and MS risk. Third, the two GWAS from European ancestry reduce the influence of population stratification. Fourth, using independent statistical methods, we proved 20 independent genetic variants to be the effective instrumental variables. Fifth, we used five MR analysis methods including MR-egger, weighted median, inverse variance weighted (multiplicative random effects), simple mode, and weighted mode. Critically, all five MR methods proved the causal association between 25OHD levels and MS risk. Lastly, our results were robust, with no obvious bias based on the different methods.

Of course, our MR study had some limitations. First, our MR analysis is only based on European ancestry. Therefore, our conclusions need to be investigated in other ancestries. Second, the GWAS dataset from IMSGC is based on clinically diagnosed MS [32]. Different diagnostic criteria may produce different results. Third, among 115 newly identified serum 25OHD genetic variants [31], only 20 25OHD genetic instrumental variables were effective and independent. Fourth, although we proved that increased serum 25OHD levels could reduce MS risk, the mechanisms by which 25OHD reduced MS risk is still unclear. Fifth, the genetically increased serum 25OHD level does not necessarily reflect the active form of vitamin D, 1,25-Dihydroxyvitamin D3 (1,25(OH)2D3), in the serum [49]. Sixth, while our MR studies may provide the strongest evidence for a causal link between vitamin D levels and the risk of MS, our study does not address the role of vitamin D on disease activity in patients with established MS. Therefore, our study must expand to explore the causal association of vitamin D levels with the activity of MS disease using other databases.

Although MR study offers several advantages, it also has many limitations [50]. These limitations include pleiotropy, low statistical power, canalization, population stratification (which is confounding by ancestry), etc. [50]. To detect and correct for pleiotropy, probably the most challenging to address, a robust method MR-egger intercept was used in our MR study. To improve the statistical power, we used the, to date, largest large-scale GWAS for serum 25OHD [31] and MS [32]. To eliminate population stratification, both 25OHD GWAS and MS GWAS were chosen from European descent. Although we try to minimize the shortcomings, maybe there are still some limitations. Thus, our results need be reproduced in randomized controlled trials (RCTs).

## Conclusions

In summary, our MR analysis strengthens evidence for the causal association between genetically increased serum 25OHD levels and reduced MS in European people.

## Supplementary Information

**Additional file 1: Table S1**. Main characteristics of 115 independent vitamin D genetic variants.

## Data Availability

The summary statistics for the genetic associations of 25OHD (GWAS ID: ebi-a-GCST90000615) and MS GWAS datasets (GWAS ID: ieu-a-1025) can be found on the ieu open gwas project at https://gwas.mrcieu.ac.uk/datasets/. The MR analysis code can be found at https://mrcieu.github.io/TwoSampleMR/articles/index.html.

## Abbreviations

25OHD: 25-Hydroxyvitamin D
MS: Multiple sclerosis
GWAS: Genome-wide association study
MR: Mendelian randomization
SNP: Single nucleotide polymorphism
IMSGC: The International Multiple Sclerosis Genetics Consortium
IVW: Inverse variance weighted
