# Supplementary material for: Mendelian randomization study updates the effect of 25-hydroxyvitamin D levels on the risk of multiple sclerosis
Source: J Transl Med. 2022 Jan 3;20:3. doi: 10.1186/s12967-021-03205-6 (PMC8722044; doi:10.1186/s12967-021-03205-6)
Supplement: Supplementary file 1 — Additional file 1: Table S1. Main characteristics of 115 independent vitamin D genetic variants. [file 12967_2021_3205_MOESM1_ESM.pdf]

Supplementary table 1. Main characteristics of 115 independent vitamin D genetic variants.

	SNP	chr	position	EA	NEA	EAF	*P* val	Beta	SE
1	rs35408430	1	17560195	T	C	0.342	5.82E-25	-0.021	0.002
2	rs2642439	1	220970499	G	A	0.685	6.18E-12	-0.015	0.002
3	rs79687284	1	214150821	C	G	0.035	3.77E-08	-0.030	0.005
4	rs12123821	1	152179152	T	C	0.048	3.71E-63	0.077	0.005
5	rs2131925	1	63025942	T	G	0.644	2.43E-27	-0.022	0.002
6	rs61816766	1	152319572	C	T	0.029	2.75E-49	0.087	0.006
7	rs6672758	1	230303512	T	C	0.801	7.16E-11	0.016	0.002
8	rs512083	1	46027355	C	T	0.461	6.56E-10	0.012	0.002
9	rs6671730	1	417580	A	G	0.434	2.25E-14	-0.015	0.002
10	rs7528419	1	109817192	G	A	0.225	1.34E-17	0.020	0.002
11	rs10908469	1	155468732	C	A	0.270	6.67E-13	0.016	0.002
12	rs7522116	1	41835685	T	C	0.566	2.58E-10	-0.013	0.002
13	rs79598313	1	27284913	T	C	0.023	1.13E-08	-0.037	0.006
14	rs1260326	2	27730940	C	T	0.607	3.95E-29	0.022	0.002
15	rs13011615	2	21274167	T	A	0.135	4.04E-09	0.017	0.003
16	rs6723486	2	58979783	T	C	0.611	4.24E-08	-0.011	0.002
17	rs7569755	2	118648261	A	G	0.291	1.21E-09	0.013	0.002
18	rs1047891	2	211540507	A	C	0.316	2.58E-09	-0.013	0.002
19	rs2012736	2	234622379	A	C	0.081	1.10E-40	-0.048	0.004
20	rs6724965	2	101440151	G	A	0.171	1.76E-10	-0.017	0.003
21	rs6547409	2	21190209	T	C	0.050	6.57E-10	0.028	0.005
22	rs58387006	2	32579999	C	A	0.222	6.33E-09	-0.014	0.002
23	rs34186890	3	141720712	G	A	0.260	2.25E-11	-0.015	0.002
24	rs2246832	3	49881134	T	A	0.501	1.87E-18	0.017	0.002
25	rs113642272	3	85579472	T	G	0.636	1.73E-21	-0.020	0.002
26	rs11458206	3	125125159	TG	T	0.272	1.18E-09	0.014	0.002
27	rs10028494	4	69970937	C	A	0.225	5.18E-33	0.028	0.002
28	rs6834488	4	88178919	T	C	0.424	1.96E-13	-0.015	0.002
29	rs4616820	4	57745481	T	C	0.465	5.32E-10	-0.012	0.002
30	rs2579309	4	72077830	A	G	0.067	6.23E-09	0.023	0.004
31	rs36102036	4	72912236	A	G	0.345	8.71E-60	0.034	0.002
32	rs1352846	4	72617775	G	A	0.291	1.00E-200	-0.193	0.002
33	rs189407772	4	100146674	G	A	0.023	1.02E-15	0.053	0.007
34	rs10084913	4	72338030	G	A	0.134	8.73E-11	0.019	0.003
35	rs4364259	4	15892159	A	G	0.202	2.23E-11	0.017	0.002
36	rs78649910	4	3482213	A	T	0.106	1.57E-09	-0.019	0.003
37	rs3890624	4	166251068	G	A	0.395	3.19E-08	0.011	0.002
38	rs1966478	5	118627319	C	T	0.693	6.92E-09	-0.012	0.002

39	rs2608984	6	131918839	T	A	0.165	2.79E-15	-0.021	0.003
40	rs72834856	6	22801858	G	T	0.072	4.86E-10	-0.024	0.004
41	rs11751024	6	32586236	A	C	0.397	3.76E-10	-0.013	0.002
42	rs9467550	6	25653401	G	A	0.105	3.36E-09	-0.019	0.003
43	rs143069752	6	40962537	A	T	0.067	1.16E-08	0.022	0.004
44	rs9476310	6	57767576	T	C	0.511	2.67E-09	0.012	0.002
45	rs1858889	7	107117447	C	A	0.502	1.38E-08	0.011	0.002
46	rs2346264	7	133536351	C	A	0.783	8.86E-10	-0.015	0.002
47	rs7784802	7	64015379	T	A	0.361	1.40E-11	0.014	0.002
48	rs17144574	7	21563471	C	T	0.234	6.73E-11	-0.015	0.002
49	rs41301394	7	75612803	T	C	0.282	7.11E-09	0.013	0.002
50	rs57459725	8	61312205	G	C	0.133	1.07E-09	-0.018	0.003
51	rs804281	8	11611865	G	A	0.584	2.48E-15	0.016	0.002
52	rs34726834	8	25889606	T	C	0.252	4.49E-11	0.015	0.002
53	rs12056768	8	116988527	G	T	0.583	3.27E-28	-0.022	0.002
54	rs9409266	9	125745042	A	G	0.861	8.74E-11	-0.018	0.003
55	rs532436	9	136149830	A	G	0.184	1.82E-11	-0.017	0.003
56	rs13284054	9	107669073	C	T	0.118	1.82E-09	0.019	0.003
57	rs4418728	10	94839724	T	G	0.452	7.54E-10	0.012	0.002
58	rs10822145	10	64934548	T	C	0.475	1.60E-10	-0.013	0.002
59	rs2297991	10	113913222	C	T	0.721	3.51E-08	0.012	0.002
60	rs3925446	10	91495322	A	G	0.199	3.68E-11	0.016	0.002
61	rs964184	11	116648917	C	G	0.868	3.37E-50	0.043	0.003
62	rs1660818	11	71028249	A	G	0.323	2.74E-14	0.016	0.002
63	rs116970203	11	14876718	A	G	0.027	1.00E-200	-0.377	0.006
64	rs12798050	11	71223256	T	C	0.830	1.00E-200	0.111	0.003
65	rs2276360	11	71169547	C	G	0.788	1.00E-200	0.111	0.002
66	rs113140528	11	14350665	T	A	0.177	3.57E-36	0.033	0.003
67	rs36037728	11	71111987	T	C	0.026	7.69E-22	-0.059	0.006
68	rs7128011	11	14462532	A	G	0.368	1.00E-200	-0.075	0.002
69	rs10766281	11	15728444	G	A	0.256	7.62E-12	0.015	0.002
70	rs575976	11	75437630	G	A	0.182	1.06E-09	0.016	0.003
71	rs33981819	11	66079361	G	T	0.461	1.20E-08	0.011	0.002
72	rs77037130	11	14467795	A	G	0.015	1.55E-17	-0.068	0.008
73	rs2847500	11	120114421	A	G	0.124	1.22E-13	-0.022	0.003
74	rs12317268	12	21352541	G	A	0.151	5.27E-14	-0.021	0.003
75	rs11182428	12	38526387	C	T	0.520	3.17E-10	-0.012	0.002
76	rs8181687	12	68661596	A	G	0.579	4.00E-09	0.012	0.002
77	rs10859995	12	96375682	C	T	0.583	1.11E-89	-0.040	0.002
78	rs73413596	12	111582630	C	T	0.074	2.89E-10	0.024	0.004
79	rs2171427	12	24822154	A	G	0.155	1.81E-08	-0.015	0.003
80	rs35014562	13	55717980		G	0.281	1.01E-08	-0.013	0.002
81	rs8018720	14	39556185	C	G	0.823	1.95E-50	-0.038	0.003
82	rs2756119	14	104001517	A	G	0.383	2.35E-09	0.012	0.002

83	rs142004400	14	50829560	C	A	0.035	6.43E-10	-0.033	0.005
84	rs62012775	15	63871274	T	A	0.176	2.77E-10	-0.016	0.003
85	rs1800588	15	58723675	T	C	0.215	1.50E-43	-0.033	0.002
86	rs1532085	15	58683366	G	A	0.615	3.62E-38	0.026	0.002
87	rs2123930	15	100231033	A	G	0.279	1.30E-10	-0.014	0.002
88	rs77924615	16	20392332	A	G	0.193	8.09E-09	-0.014	0.003
89	rs11542462	16	82033810	A	G	0.134	5.79E-16	-0.023	0.003
90	rs8063565	16	30883965	C	G	0.734	2.68E-08	0.012	0.002
91	rs11076175	16	57006378	G	A	0.178	9.46E-21	0.024	0.003
92	rs34177108	16	89893375	A	C	0.268	3.96E-08	-0.012	0.002
93	rs2952289	17	66464414	T	C	0.798	3.22E-13	0.018	0.002
94	rs2659007	17	79217478	A	G	0.451	9.75E-09	0.011	0.002
95	rs61003750	17	40793128	C	G	0.307	1.85E-08	-0.012	0.002
96	rs12949853	17	7570878	A	G	0.807	1.23E-08	0.014	0.003
97	rs4121823	18	47144223	A	T	0.845	1.33E-11	-0.019	0.003
98	rs8091117	18	28919794	A	C	0.065	2.34E-10	-0.025	0.004
99	rs2037511	18	61366207	A	G	0.166	1.07E-10	0.017	0.003
100	rs12462826	19	11955767	A	G	0.369	1.52E-10	-0.013	0.002
101	rs429358	19	45411941	C	T	0.152	1.76E-17	-0.023	0.003
102	rs212100	19	48376995	C	T	0.836	7.83E-136	-0.066	0.003
103	rs3814995	19	36342212	T	C	0.312	2.33E-09	-0.013	0.002
104	rs200210321	19	19393890	AG	A	0.073	9.84E-26	0.040	0.004
105	rs142158911	19	11190534	A	G	0.115	7.17E-18	0.027	0.003
106	rs11606	19	54658102	G	C	0.425	2.77E-08	0.011	0.002
107	rs62115743	19	51514874	T	C	0.082	3.21E-14	0.027	0.004
108	rs1841850	20	52718179	C	A	0.113	2.31E-19	0.028	0.003
109	rs2207132	20	39142516	A	G	0.033	1.96E-12	-0.039	0.005
110	rs8121940	20	52742306	G	C	0.195	1.69E-52	-0.038	0.002
111	rs2229742	21	16339172	C	G	0.103	5.90E-15	-0.025	0.003
112	rs2074735	22	31535872	C	G	0.064	4.00E-12	0.028	0.004
113	rs115621755	22	50853134	T	C	0.327	1.53E-09	-0.013	0.002
114	rs6003465	22	23365501	C	T	0.332	1.19E-08	-0.012	0.002
115	rs5965373	X	66409433	G	T	0.857	1.41E-08	0.013	0.002

Beta is the regression coefficient based on the vitamin D raising allele (effect allele); SNP: single-nucleotide polymorphism; EA: effect allele; NEA: non-effect allele; EAF: effect allele frequency; SE: standard error.
